# Mapping the Network Structure of Psychosocial Symptoms and School Well-Being Across Gender in Secondary School Students

**DOI:** 10.3390/ejihpe16040054

**Published:** 2026-04-16

**Authors:** Philippos Zdoupas

**Affiliations:** Department for Special Educational Needs, University of Duisburg-Essen, 45217 Essen, Germany; philippos.zdoupas@uni-due.de; Tel.: +49-201-183-7652

**Keywords:** gender differences, mental health, network analysis, psychosocial symptoms, school well-being

## Abstract

Gender differences in the prevalence of psychosocial problems during adolescence are well established, with girls reporting higher internalizing symptoms and boys higher externalizing symptoms. However, it remains unclear whether these differences extend beyond symptom levels to the structural organization linking psychosocial problems and school well-being (SWB). The present study examined gender-specific network structures comprising internalizing symptoms, externalizing symptoms, and six dimensions of SWB in a sample of 949 secondary school students in Germany (Grades 8–10; 50.6% boys, 49.4% girls). Partial correlation networks were estimated separately for boys and girls using EBIC-regularized graphical models, followed by network comparison tests and centrality analyses. Results indicated no significant differences in global strength, network structure, or individual edges between genders, suggesting a largely shared network structure. Across both networks, internalizing symptoms, particularly symptoms of anxiety and depression, emerged as central and bridging nodes connecting psychosocial problems with multiple dimensions of SWB. Externalizing symptoms showed minor descriptive differences in prominence but did not alter the overall structural pattern. These findings indicate that gender differences in adolescent mental health may reflect differences in symptom intensity rather than fundamentally distinct psychosocial systems, suggesting common structural patterns underlying SWB across gender.

## 1. Introduction

Psychosocial problems in childhood and adolescence represent one of the most significant challenges for health systems and educational institutions. Meta-analytical and large-scale epidemiological studies indicate that approximately 15–25% of young people meet the criteria for at least one mental disorder at any given time (e.g., Castaldelli-Maia et al., 2025; Polanczyk et al., 2015). Many of these conditions emerge before the age of 14 and frequently remain unrecognized and untreated (Merikangas et al., 2010; Sacco et al., 2024). Importantly, prevalence rates differ by symptom type. Internalizing problems (e.g., anxiety and depression) are particularly widespread in adolescence, whereas externalizing problems (e.g., aggression, conduct problems, and hyperactivity) are more commonly observed in childhood and tend to decline with increasing age (Ihle & Esser, 2002; Otto et al., 2021). These patterns underscore that psychosocial vulnerability in childhood and adolescence is neither rare nor homogeneous, but rather characterized by heterogeneous symptom profiles. A further consistent finding concerns gender differences in prevalence rates. Across countries and socio-demographic contexts, girls are more likely to report internalizing problems, whereas boys more frequently exhibit externalizing problems (Campbell et al., 2021; Castaldelli-Maia et al., 2025; Riecher-Rössler, 2017; Yoon et al., 2023). These differences tend to intensify during adolescence and have been linked to biological maturation processes, gender intensification mechanisms, and gendered patterns of emotional socialization and coping (Cela-Bertran et al., 2024; Esteban-Gonzalo et al., 2020; Laubenstein & Zdoupas, 2026; Lewis et al., 2018). Thus, gender differences in psychosocial functioning are among the most robust findings in developmental psychopathology.

Given the high prevalence and gendered distribution of psychosocial problems, understanding their implications within central developmental contexts is essential. One such context is school. Schools are not only academic institutions but also pursue broader educational aims encompassing emotional, social, and personal development (Eckert et al., 2025; Schnell et al., 2025). Contemporary educational frameworks emphasize that fostering students’ social and emotional skills is foundational to their overall development (OECD, 2023; UNESCO, 2025). Within these frameworks, school-related well-being (SWB) has gained increasing attention as a multidimensional construct. Conceptually, it encompasses interrelated affective, social, and competence-related dimensions, including students’ emotional experiences in school, their perceived belonging and relationships, and their satisfaction with school as well as their perceived academic competence (Hossain et al., 2023; Kullmann et al., 2023; Schnell et al., 2025; Zdoupas et al., 2025). Empirical evidence supports this multidimensional structure and indicates that SWB is associated with academic engagement, motivation, attendance, longer-term psychosocial adjustment, and family-related contextual factors such as parental involvement (e.g., Obermeier et al., 2026; Schlesier & Obermeier, 2025; Steinmayr et al., 2018; Tobia et al., 2019). Consequently, SWB constitutes a key indicator of how effectively schools fulfill their broader educational objectives (Bücker et al., 2018). In this context, a growing body of research identifies psychosocial problems as central individual correlates of SWB. Across studies, both internalizing and externalizing symptoms are systematically associated with lower levels of SWB (Arslan & Coşkun, 2020; Arslan & Renshaw, 2018; Kim & Choe, 2022; Tobia et al., 2019; van Loon-Dikkers et al., 2025). Notably, internalizing symptoms tend to exhibit particularly strong and pervasive negative associations, suggesting heightened vulnerability among students reporting elevated levels of anxiety and depression (Kaplan, 2017; Serrão et al., 2024; Zdoupas & Laubenstein, 2026; Zdoupas et al., 2025). Although gender differences in the prevalence of psychosocial symptoms are well established, the role of gender in their association with SWB remains insufficiently understood. Evidence on gender differences in SWB itself is mixed and appears to depend on how the construct is operationalized. Studies relying on global indicators often report higher levels among girls, whereas analyses of specific subdimensions partly indicate advantages for boys (e.g., Liu et al., 2016; Markus et al., 2022; Hascher & Hagenauer, 2018). These inconsistencies likely reflect differences in dimensional focus and measurement (Kaye-Tzadok et al., 2017; Kiuru et al., 2020). However, most research has centered on mean-level comparisons or predictive effects, leaving open whether the relational structure linking psychosocial symptoms and dimensions of SWB differs across gender. Distinguishing between differences in symptom intensity and differences in structural organization is conceptually important. Higher prevalence of internalizing symptoms among girls, for instance, does not necessarily imply that the pattern of associations between internalizing symptoms and distinct SWB components differs structurally between boys and girls. To address this distinction, analytical approaches are needed that move beyond regression-based models and allow for the examination of relational network structures among multiple interrelated components.

Network analysis offers such a framework (Borsboom et al., 2021). By conceptualizing symptoms and SWB dimensions as interconnected nodes within a system, network models estimate partial associations and identify central as well as bridging elements linking symptom domains with school-related experiences. Unlike regression-based approaches, which typically model unidirectional effects of one variable on another while holding others constant, network analysis estimates a system of pairwise conditional associations among all variables simultaneously. This allows for the identification of central nodes—variables that are most strongly connected within the system—as well as bridge variables that link distinct symptom clusters. Crucially, network comparison procedures permit formal tests of whether the structure of pairwise conditional associations differs across groups (van Borkulo et al., 2023), complementing latent variable approaches such as SEM, which assume an underlying factor structure, by modeling the association system directly at the observed variable level without such assumptions. This makes it possible to examine whether gender differences in psychosocial prevalence extend to differences in the organization of symptom-well-being networks. Emerging network studies in adolescent populations have reported structural invariance across gender despite mean-level symptom differences (Angulo-Salas et al., 2026), yet it remains unclear whether this pattern extends to the specific relational structure linking psychosocial problems with multidimensional SWB. The present study addresses this gap by estimating and comparing gender-specific networks comprising internalizing symptoms, externalizing symptoms, and multiple dimensions of SWB in a large sample of secondary school students in Germany. Given the limited prior work on this specific configuration of variables, the present study is exploratory in nature, aiming to map rather than confirm structural relationships within the symptom-SWB network. Three research questions guided the analyses:

**RQ_1_:**  *Do boys and girls show differences in the overall strength or structural organization of their symptom-SWB networks, beyond mean-level symptom differences?*

**RQ_2_:**  *Which symptoms and SWB dimensions are most central within each network, and are these patterns comparable across gender?*

**RQ_3_:**  *Which symptoms connect psychosocial problem domains with SWB dimensions, and does this bridging role differ between boys and girls?*

By integrating epidemiological evidence on psychosocial prevalence with a multidimensional understanding of SWB, the study aims to clarify whether observed gender differences reflect distinct relational mechanisms or variations within a largely shared psychosocial structure.

## 2. Materials and Methods

### 2.1. Participants and Procedure

Data were collected as part of the WINS project (“Well-being in Inclusive Schools”) (Zdoupas & Laubenstein, 2024). Schools were recruited through a regional headteacher conference at which inclusive secondary schools were informed about the study and invited to participate voluntarily. Of the 12 inclusive secondary schools represented, seven agreed to participate. A total of 1002 questionnaires were distributed across these schools, of which 976 were retained for analysis after excluding cases with missing values on psychosocial problems or SWB scales (*n* = 26; 2.6% of distributed questionnaires). Given the low proportion of missing data, listwise deletion is unlikely to have introduced systematic bias into the analyses. The resulting sample comprised *N* = 976 students in grades 8–10 attending seven inclusive secondary schools in North Rhine-Westphalia, Germany. The sample distribution was balanced across grade 8 (39%), 9 (31%), and 10 (30%). Individual age was not assessed; grade membership served as a proxy for developmental stage. Ten percent of the students had formally diagnosed special educational needs. The sample was nearly evenly distributed across gender (49.2% male, 48.2% female). For gender-based network comparisons, analyses were restricted to students identifying as male or female. Due to insufficient subgroup size for stable network estimation, students identifying as non-binary (*n* = 15) or with missing gender information (*n* = 11) were excluded. After listwise deletion, the final analytic sample comprised *N* = 949 students (boys: *n* = 480; girls: *n* = 469). Pseudonymized paper-and-pencil questionnaires were administered during regular class periods by trained project staff following parental informed consent and students’ written assent.

### 2.2. Measures

**SWB** was measured using the Questionnaire for the Assessment of School Well-Being in Inclusive Classes (Kullmann et al., 2023). The questionnaire consists of 24 items allocated to six domains: Attitudes Toward School (ATS; e.g., *“I feel comfortable at school”*), Affinity to Class (ATC; e.g., *“I enjoy being with my classmates”*), Academic Self-Esteem (ASE; e.g., *“I have no problems meeting the demands at school”*), Concerns in School (CIS; e.g., *“In the past weeks, I have been worried about how to manage upcoming tasks”*), Social Problems in School (SPS; e.g., *“In the past weeks, I felt like an outsider in my class”*), and Somatic Complaints in School (SCS; e.g., *“In the past weeks, I had stomach aches because of school”*). Responses are provided on a six-point Likert-type scale ranging from 0 (“does not apply at all”) to 5 (“fully applies”). For analytic purposes, the CIS, SPS, and SCS scales were recoded so that higher scores uniformly indicate higher levels of SWB. Internal consistency estimates (Cronbach’s α and McDonald’s ω) for the present sample were acceptable to good across all subscales (ATS: α=0.84, ω=0.84; ATC: α=0.79, ω=0.80; CIS: α=0.70, ω=0.73; SPS: α=0.76, ω=0.78; SCS: α=0.81, ω=0.82) except for ASE (α=0.63, ω=0.64).

**Psychosocial problems** were assessed using the self-report version of the Strengths and Difficulties Questionnaire (SDQ-S; Goodman, 2005). Four symptom domains were considered: Emotional Symptoms (ES; e.g., *“I worry a lot”*) and Peer Problems (PP; e.g., *“I usually keep to myself”*) representing internalizing problems, and Conduct Problems (CP; e.g., *“I get very angry and lose my temper”*) and Hyperactivity/Inattention (HI; e.g., *“I am restless, I cannot stay still for long”*) representing externalizing problems. Items are rated on a 3-point scale (0 = “does not apply at all”, 1 = “somewhat applies”, 2 = “certainly applies”). Higher scores indicate greater symptom severity. Internal consistency estimates indicated mixed reliabilities for ES (α=0.74, ω=0.74), HI (α=0.72, ω=0.73), CP (α=0.68, ω=0.68) and PP (α=0.57, ω=0.58). These values are consistent with findings from validation studies of the German self-report SDQ, in which the PP and CP subscales typically show moderate to low reliability, attributable to their heterogeneous item content and limited subscale length (Altendorfer-Kling et al., 2007; Boe et al., 2016; Lohbeck et al., 2015; Rønning et al., 2004). As network analysis operates on covariance structures and partial correlations rather than composite scores, all four subscales were retained in the analyses despite the suboptimal reliability of the latter two subscales.

**Gender** was assessed via self-report and is therefore operationalized as a socially constructed identity category rather than a strictly biological variable. Consistent with this, the term “gender” is used throughout, while acknowledging that the binary format does not fully capture the multidimensional nature of gender identity (Cela-Bertran et al., 2024).

### 2.3. Data Analyses

Network analysis was conducted using the *qgraph* (Epskamp et al., 2012), *ggplot2* (Wickam, 2016) and *NetworkComparisonTest* (van Borkulo et al., 2023) packages in R (version 4.5.2). The analytical strategy proceeded in five steps:

(1) To estimate the conditional association structure among all variables separately for each gender group, EBIC-regularized partial correlation networks were estimated separately for boys (*n* = 480) and girls (*n* = 469) using the graphical lasso (glasso) algorithm with Extended Bayesian Information Criterion (EBIC; γ=0.5). The network comprised 10 nodes: four SDQ symptom subscales (ES, PP, CP, HI) and six SWB dimensions (ATS, ATC, ASE, CIS, SPS, SCS). Edge weights represent regularized partial correlations between nodes, controlling for all other variables in the network. All reported edge weights are standardized regularized partial correlations.
(2) To formally test whether the two gender-specific networks differ in their overall connectivity or edge-level structure, network comparison tests (NCT) were performed. Three invariance tests were conducted: (a) global strength invariance (sum of absolute edge weights), (b) structural invariance (maximum difference in edge weights), and (c) edge-specific differences (FDR-corrected) (van Borkulo et al., 2023). NCT used permutation-based procedures (2000 iterations) to generate null distributions.
(3) To identify which symptoms serve as connectors between psychosocial problem domains and SWB dimensions, bridge centrality (1-step expected influence; Jones et al., 2021) was computed to identify symptoms connecting psychosocial problems with SWB domains. Communities were defined as Internalizing (ES, PP), Externalizing (CP, HI), and SWB (six dimensions).
(4) To determine the most structurally prominent nodes within each network, strength analyses were computed, comprising strength centrality (sum of absolute edge weights) and expected influence (1-step; sum of signed edge weights). While strength centrality identifies highly connected nodes regardless of edge direction, expected influence accounts for connection valence (positive vs. negative associations), indicating whether nodes function as amplifiers or suppressors within the system (Robinaugh et al., 2016).
(5) To assess the reliability of edge weight and centrality estimates, network stability and global properties were assessed via non-parametric bootstrap (*n* = 1000) and case-dropping procedures to calculate correlation stability (CS) coefficients for edge weights and centrality indices (Epskamp et al., 2018). CS > 0.25 indicates acceptable stability. Global network density was calculated as the proportion of non-zero edges relative to all possible connections.

Prior to mean-level comparisons, measurement invariance of the SWB instrument across gender was examined using multigroup confirmatory factor analysis (CFA) with a robust MLR estimator and Satorra-Bentler corrected chi-square difference tests (Satorra & Bentler, 2010). Model fit was evaluated using CFI, RMSEA, and SRMR, with changes in CFI (ΔCFI ≤ 0.01) serving as the primary criterion for invariance (Chen, 2007; Cheung & Rensvold, 2002). In addition, descriptive statistics and independent-samples t-tests were conducted to examine mean-level gender differences in psychosocial symptoms and SWB dimensions. Effect sizes were calculated using Cohen’s *d*. This step was included to differentiate between differences in symptom intensity and differences in relational structure.

## 3. Results

### 3.1. Measurement Invariance and Mean-Level Gender Differences

Prior to mean-level analyses, measurement invariance of the SWB instrument across gender was tested. The configural baseline model showed acceptable fit (CFI = 0.898, RMSEA = 0.052, SRMR = 0.056). The metric model with constrained factor loadings did not show a significant deterioration in fit ($\Delta \chi^{2}(18)=19.12$, p=0.384; ΔCFI=0.000), supporting metric invariance. The scalar model with additionally constrained intercepts yielded a significant chi-square difference ($\Delta \chi^{2}(18)=44.02$, p<0.001), though the change in CFI remained within acceptable bounds (ΔCFI=−0.005; Chen, 2007; Cheung & Rensvold, 2002). By conservative criteria, full scalar invariance was not achieved, while metric invariance was established. This supports the comparability of covariance structures (i.e., network edges) across gender, while direct latent mean comparisons should be interpreted with caution. Table 1 presents means, standard deviations, and gender comparisons for all variables. The following text highlights key differences by effect size. Consistent with established epidemiological patterns, girls reported significantly higher ES than boys, reflecting a large effect (d=−0.99, p<0.001). No significant gender difference emerged for HI or PP. Boys reported higher CP than girls, though the effect size was small (d=0.15, p=0.025; see Table 1).

Among SWB dimensions, boys reported more positive levels on CIS and SCS than girls, both with medium-to-large effect sizes (d=0.70 for both). Conversely, girls reported lower ATC than boys (d=0.25, p<0.001). No significant gender differences were observed for ATS, ASE, or SPS.

These mean-level differences confirm that gender differences in symptom intensity and SWB are present in the current sample, providing the empirical foundation for examining whether such intensity differences extend to structural network differences.

### 3.2. Step 1: Network Estimation

**Figure 1:**
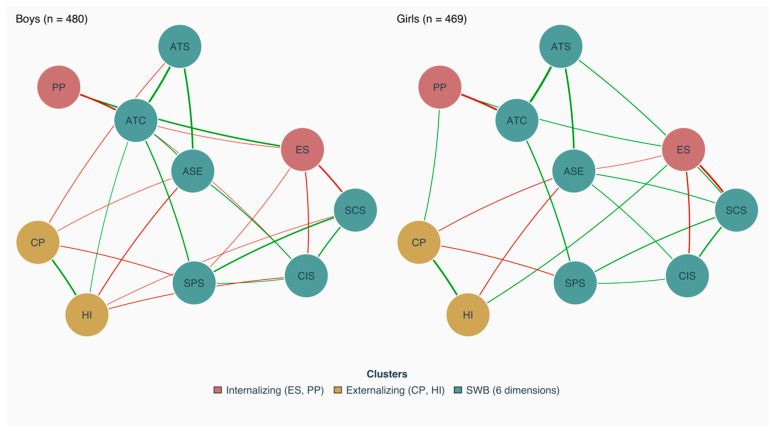
Network Structures. Note. ES = Emotional Symptoms; PP = Peer Problems; CP = Conduct Problems; HI = Hyperactivity/Inattention; ATS = Attitudes Toward School; ATC = Affinity to Class; ASE = Academic Self-Esteem; CIS = Concerns in School; SPS = Social Problems in School; SCS = Somatic Complaints in School. Green edges indicate positive associations; red edges indicate negative associations. Edge thickness reflects the magnitude of the association.

Figure 1 displays the estimated networks for boys (*n* = 480) and girls (*n* = 469). Both networks exhibited high density (boys: 0.78; girls: 0.73), indicating that 78% and 73% of all possible edges were retained after regularization, respectively. This suggests a highly interconnected system where most psychosocial symptoms and well-being dimensions maintain direct conditional associations.

Within the internalizing cluster, ES show the strongest connections overall, with particularly pronounced negative edges to CIS (boys: r=−0.16; girls: r=−0.23) and SCS (boys: r=−0.28; girls: r=−0.35), indicating that elevated emotional distress is closely associated with school-related worries and psychosomatic complaints. The connection between ES and PP was notably stronger among boys (r=0.26) than girls (r=0.09), suggesting that emotional and social difficulties are more tightly coupled within the boys’ network. PP show weaker but consistent connections to SWB dimensions, most notably to ATC (boys: r=−0.32; girls: r=−0.37). Within the externalizing cluster, HI maintains broader connections to SWB nodes compared to CP, particularly in the boys’ network (e.g., HI-ASE: boys r=−0.16, girls r=−0.14; HI-CIS: boys r=−0.10, girls r=0.00), whereas CP shows more circumscribed associations.

Among SWB nodes, ATC shows the highest number of connections, linking to both internalizing and externalizing symptom nodes in both networks. The strongest within-domain edge was observed between ATS and ATC (boys r=0.36; girls r=0.41), reflecting a close association between general school attitudes and peer belonging across both groups. Several further edges between SWB dimensions are present in both networks (e.g., ATS-ASE: boys r=0.30, girls r=0.30; CIS-SCS: boys r=0.20, girls r=0.23), suggesting coherent within-domain associations independent of gender.

Overall, both networks reflect a coherent pattern in which internalizing symptoms, particularly ES, are most strongly embedded within the symptom-well-being system, while ATC serves as the most connected well-being node, with descriptive but non-significant differences emerging primarily for externalizing symptoms. Descriptive differences in individual edge weights between the two networks should be interpreted with caution, as they were not formally tested at the edge level and may reflect sampling variability rather than systematic structural distinctions.

### 3.3. Step 2: Network Comparison

Network comparison tests reveal no significant structural differences between boys and girls. Global strength invariance is not significant (S=0.056, p=0.894), indicating equivalent overall connectivity density. Structural invariance is not significant (M=0.162, p=0.093), suggesting similar network structures. Although the structural invariance test did not reach significance, the observed maximum edge difference (M=0.170) was of moderate magnitude, suggesting that the absence of significant differences is not solely attributable to insufficient statistical power. Edge-specific comparisons reveal no significant differences after FDR correction (0/45 edges), demonstrating that individual connections between specific symptoms and SWB dimensions do not differ statistically across genders. These findings support the interpretation of a largely shared psychosocial network structure between boys and girls. Network stability analysis revealed good correlation stability for edge weights in both networks (boys: CS=0.67; girls: CS=0.75), exceeding the recommended threshold of 0.25. Strength centrality showed acceptable stability (boys: CS=0.36; girls: CS=0.67).

Taken together with the mean-level differences reported above, these results indicate that boys and girls differ primarily in the *intensity* of ES and selected SWB dimensions (notably CIS and SCS), but not in the *conditional association structure* linking these variables.

### 3.4. Step 3: Bridge Centrality

**Figure 2:**
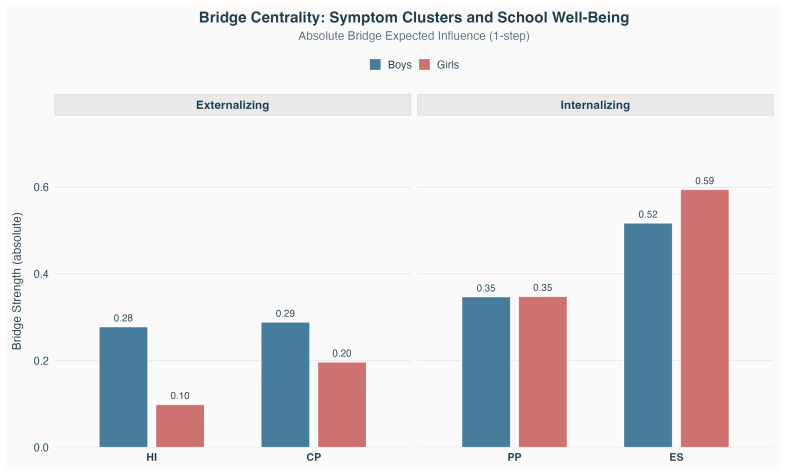
Bridge Centrality.

Figure 2 illustrates bridge centrality values for symptom nodes, quantifying the extent to which symptoms connect their respective domains with the SWB domain. ES exhibit the strongest bridge centrality for both boys (|EI|=0.52) and girls (|EI|=0.59), indicating that this node most strongly connects the internalizing domain with SWB dimensions. The magnitude of this bridging function is comparable across genders, suggesting that ES serve as a primary pathway linking internalizing problems to SWB for all students. PP show equivalent bridge centrality across genders (|EI|=0.35).

Among externalizing symptoms, HI demonstrates substantially higher bridge centrality for boys (|EI|=0.28) compared to girls (|EI|=0.10). This suggests that inattentive and hyperactive behaviors serve as more prominent connectors between externalizing problems and SWB impairments for boys. However, this difference was not formally tested at the node level and should be interpreted as a descriptive pattern only. CP show comparable bridge values across genders (boys: |EI|=0.29; girls: |EI|=0.20).

Overall, bridge centrality analyses highlight ES as the most consistent cross-domain connector in both networks, while differences in the bridging role of externalizing symptoms across gender remain descriptive and statistically unconfirmed.

### 3.5. Step 4: Centrality Patterns

Table 2 displays strength centrality indices. ATC emerged as the most central node overall for both boys (C=1.16) and girls (C=1.07), underscoring the structural prominence of peer relationships and classroom belonging. Among symptom nodes, ES showed the highest centrality (boys: C=0.87; girls: C=0.92), confirming their position as core elements within the psychosocial problem domain.

Gender differences in symptom centrality are observed for externalizing problems. HI is more central for boys (C=0.77) than girls (C=0.66), whereas CP show higher centrality for girls (C=0.70) compared to boys (C=0.58). These patterns suggest that different externalizing symptoms occupy differential positions within boys’ and girls’ psychosocial networks. However, as these differences were not statistically significant at the network level, they should be interpreted as descriptive tendencies rather than confirmed structural distinctions.

Taken together, centrality analyses converge with bridge centrality findings in identifying ES and ATC as the most structurally prominent nodes across both gender-specific networks.

**Figure 3:**
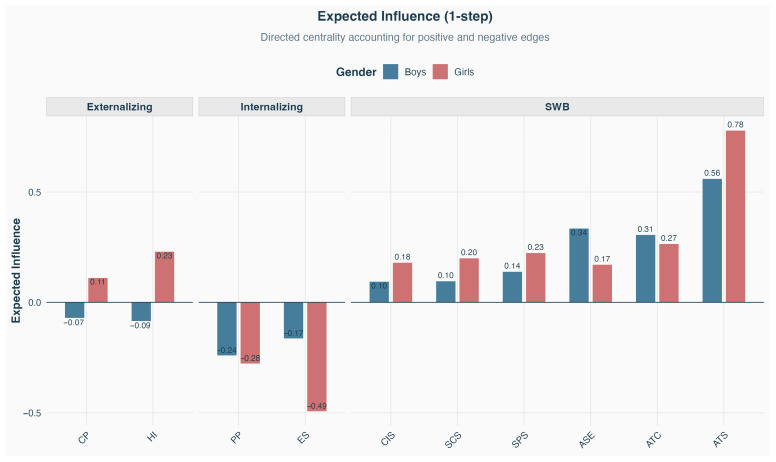
Expected Influence by Gender (Bars represent the sum of edge weights connected to each node. Higher absolute values indicate greater overall connectivity).

Figure 3 displays expected influence (1-step) values. ATC shows a positive expected influence in both networks (boys: 0.307; girls: 0.266), reflecting its role as a hub connecting multiple domains. ES exhibits negative expected influence (boys: −0.165; girls: −0.494), indicating predominantly negative associations with well-being dimensions. Notably, HI shows divergent patterns: negative influence for boys (−0.086) but positive for girls (0.231).

## 4. Discussion

The present study examined whether well-established gender differences in the prevalence of psychosocial problems extend to differences in the network structure linking psychosocial symptoms and SWB. Using psychometric network analysis, psychosocial symptoms and well-being dimensions were modeled as nodes within a system of pairwise conditional associations, estimated as regularized partial correlations. Four main findings emerge. The first finding addresses mean-level differences as a baseline. The second and third findings directly answer ${RQ}_{1}$ regarding structural invariance. The third and fourth findings address ${RQ}_{2}$ and ${RQ}_{3}$ concerning centrality and bridging roles.

*First, boys and girls differ substantially in the intensity of Emotional Symptoms (ES) and selected SWB dimensions, but not in the conditional association structure linking these variables.* Mean-level analyses revealed a large gender difference for ES (d≈1.0), and medium-to-large effects for Concerns in School (CIS) and Somatic Complaints (SCS) (d≈0.70), confirming robust intensity-based disparities consistent with prior epidemiological evidence (Castaldelli-Maia et al., 2025; Riecher-Rössler, 2017; Yoon et al., 2023). At the same time, neither global strength nor structural invariance tests reached significance, and no individual edge differed after FDR correction. This coexistence of pronounced mean-level differences and structural invariance highlights an important dissociation: gender differences in symptom magnitude do not necessarily translate into differences in how symptoms and well-being dimensions are conditionally related. Gender differences thus appear to be a matter of intensity rather than of distinct psychosocial network organization. This pattern aligns with recent network evidence from adolescent samples in other cultural contexts. Angulo-Salas et al. (2026), examining resilience, happiness, and mental health indicators in Peruvian secondary school students, similarly found no significant structural or global strength differences between males and females despite meaningful mean-level disparities—a pattern interpreted as reflecting differences in node activation intensity rather than distinct network architectures. The present findings extend this emerging pattern to a school-specific well-being framework in a European inclusive education context, suggesting that the dissociation between symptom intensity and network organization may represent a robust transdiagnostic finding across diverse cultural and institutional settings.

*Second, internalizing symptoms—particularly depression and anxiety (ES)—function as the most central and cross-domain bridging elements within both networks.* ES showed the highest strength centrality among symptom nodes and the strongest bridge expected influence values across both gender groups, linking internalizing problems with school-related worries and somatic complaints. Affinity to Class (ATC) emerged as the most central node overall, underscoring the structural importance of peer relationships and classroom belonging for SWB. These findings extend prior regression-based evidence on the negative associations between internalizing symptoms and SWB (Kaplan, 2017; Serrão et al., 2024; Zdoupas et al., 2025) by showing that internalizing symptoms are not merely associated with lower SWB but occupy central positions within a multivariate dependency structure and do so comparably for boys and girls.

*Third, descriptive differences in the role of externalizing symptoms across gender emerged, but these patterns did not reach statistical significance and should be interpreted with caution.* Hyperactivity/Inattention (HI) showed somewhat stronger bridge connections and higher centrality among boys, while Conduct Problems (CP) appeared relatively more prominent among girls. As these differences were not confirmed at the edge or network level, they may reflect sampling variation rather than stable structural distinctions. Substantively, the comparatively stronger embeddedness of HI within the boys’ network may reflect the heightened visibility of hyperactive and inattentive behaviors in classroom settings. Teachers tend to perceive and respond to ADHD-related symptoms differently depending on student gender, with boys’ attentional and regulatory difficulties more readily recognized and more broadly implicated in academic and social functioning (Olsson, 2023). This differential visibility may contribute to stronger conditional associations between HI and multiple SWB dimensions in boys’ networks. Among girls, the comparatively more prominent position of CP within the network is theoretically noteworthy given the lower overall prevalence of conduct-related problems in this group. This pattern may reflect the social dynamics underlying conduct-related behaviors among girls: it has been theorized that such behaviors can emerge as adaptive responses to hegemonic masculinity norms within peer groups, where girls may adopt dominant or aggressive behaviors as a strategy to gain social status and navigate peer hierarchies (Laubenstein & Zdoupas, 2026). These socially embedded dynamics may render conduct-related symptoms particularly salient within the school context and thus more broadly linked to school-related well-being dimensions. These observations warrant replication in larger and more diverse samples before practical conclusions are drawn.

*Fourth, the convergence of centrality and bridge centrality findings points to ES and ATC as shared structural anchors of the psychosocial system, with implications for school-based support.* In line with multidimensional models of SWB (Hascher, 2004; Hossain et al., 2023; Kullmann et al., 2023; Schnell et al., 2025) and prior studies linking psychosocial problems to lower SWB (Arslan & Coşkun, 2020; Kim & Choe, 2022; Tobia et al., 2019; van Loon-Dikkers et al., 2025), the present findings suggest that emotional distress and social belonging occupy structurally prominent positions in students’ school experiences. Because these nodes are central across both gender groups, approaches that address emotional symptoms and peer relationships may be structurally informed by these findings for all students, regardless of gender. In line with educational frameworks emphasizing social and emotional functioning (OECD, 2023), this points toward shared rather than gender-differentiated support structures as a reasonable starting point. This has particular relevance for inclusive school settings, where support structures must be feasible across heterogeneous student populations without requiring individualized gender-differentiated designs. Network-informed approaches that prioritize structurally central nodes—such as emotional symptom reduction and peer belonging—may offer a more precise and resource-efficient basis for school-based mental health programming than broad well-being curricula centered on generic positive affect promotion (Angulo-Salas et al., 2026). Given the shared network architecture observed across gender, such approaches appear structurally justified for all students, independent of symptom prevalence differences. In inclusive education contexts specifically, embedding SWB promotion within multi-tiered prevention frameworks, such as Response-to-Intervention models, may provide a practical implementation structure: universal screening at Tier 1 can identify students with elevated emotional symptoms or low peer belonging at an early stage, while targeted and individualized supports at Tiers 2 and 3 can be allocated based on symptom profiles and observed SWB trajectories rather than administrative labels (Zdoupas & Laubenstein, 2026). This approach is consistent with preventive and equity-oriented frameworks of support that prioritize students’ psychosocial needs over categorical classifications (Zdoupas, 2024).

From a methodological standpoint, the study illustrates how network analysis can sharpen the distinction between differences in symptom levels and differences in how symptoms are interconnected. By combining network estimation with formal invariance testing, the present study complements regression-based research and demonstrates that symptom-well-being associations can remain structurally stable across demographic groups even when mean levels differ considerably. Cross-sectional network models estimate conditional associations and allow the identification of central and bridging variables without relying on latent variable assumptions (Borsboom et al., 2021).

Several limitations remain. The cross-sectional design means that estimated networks reflect between-person associations at a single time point and do not permit conclusions about causal direction or within-person dynamics (Borsboom et al., 2021). It remains unclear whether ES contribute to reduced well-being, whether lower well-being amplifies symptom levels, or whether both processes unfold simultaneously. Reliance on student self-report, while appropriate for capturing subjective school experiences (Hascher, 2004), introduces the risk of common method bias: associations between self-reported symptoms and self-reported SWB dimensions may be inflated due to shared measurement variance rather than reflecting true covariation. The absence of multi-informant data (e.g., teacher or parent ratings) limits the extent to which this bias can be assessed or corrected. The exclusion of non-binary students, though methodologically necessary due to insufficient subgroup size, limits the generalizability of the findings, and the binary operationalization of gender does not fully reflect its multidimensional and socially constructed nature (Cela-Bertran et al., 2024). Regarding measurement, the three-point SDQ response format may yield lower reliability than extended Likert scales (Lozano et al., 2008), and evidence on its measurement invariance across gender is mixed (Boe et al., 2016; Rønning et al., 2004), potentially limiting strict cross-gender comparability. Regarding the SWB instrument, multigroup CFA indicated metric but not full scalar invariance across gender (ΔCFI=−0.005), meaning that while the factor structure and loadings are equivalent, some item intercepts differ. This supports the comparability of covariance structures and network edges across gender, but implies that observed mean-level differences on SWB dimensions should be interpreted with caution rather than as reflecting true latent mean differences. More broadly, psychometric network analysis itself remains subject to ongoing methodological debate, including concerns about model selection, edge stability, and the interpretation of centrality indices (Neal et al., 2022). In particular, centrality indices derived from cross-sectional networks do not imply causal influence or intervention targets—a node that is central in a between-person network may not be the most effective point of intervention at the individual level. The present findings should therefore be understood as structured descriptions of association patterns rather than definitive representations of underlying psychological systems. As an exploratory study, the present findings should be understood as hypothesis-generating rather than confirmatory, and replication in independent samples is warranted before firm conclusions are drawn.

Future research should move beyond cross-sectional designs. Longitudinal studies would allow examination of how symptoms and SWB mutually influence one another over time and whether relational structures shift across developmental stages. Complementing quantitative network approaches with qualitative and student-centered methods could further illuminate the subjective experience of psychosocial challenges in school and inform more responsive support structures (Vergara & Taja-on, 2025). Incorporating contextual variables such as teacher support, peer climate, or classroom characteristics could further illuminate how school environments shape the symptom-well-being interplay. Expanding samples to include larger and more gender-diverse populations would enable analyses beyond binary categories and allow more rigorous tests of network stability across contexts.

## 5. Conclusions

Although gender differences in the prevalence of psychosocial symptoms are well established, the present findings indicate that the network structure linking internalizing symptoms, externalizing symptoms, and multidimensional SWB is largely shared between boys and girls. Emotional Symptoms (ES) and Affinity to Class (ATC) emerge as the most structurally prominent nodes within this system: the former as the primary connector between internalizing problems and SWB, the latter as the most central hub within the SWB domain. Taken together, the results suggest that gender differences are more apparent in symptom levels than in the underlying patterns of association between psychosocial problems and SWB, pointing toward common structural mechanisms in students’ school experiences. These shared structures imply that support approaches that address emotional distress and peer belonging may be relevant for both boys and girls, rather than requiring fundamentally gender-differentiated intervention frameworks.

## Figures and Tables

**Table 1:** Means, Standard Deviations, and Gender Comparisons.

Variables	Boys M (SD)	Girls M (SD)	t (df)	*p*-Value	Cohen’s *d*
ES	0.45 (0.38)	0.89 (0.52)	−15.19 (859)	<0.001	−0.99
PP	0.56 (0.40)	0.58 (0.35)	−0.83 (934)	0.409	−0.05
CP	0.44 (0.34)	0.39 (0.36)	2.24 (942)	0.025	0.15
HI	0.73 (0.46)	0.73 (0.48)	0.02 (943)	0.985	0.00
ATS	2.33 (1.19)	2.43 (1.18)	−1.31 (947)	0.191	−0.08
ATC	3.48 (1.07)	3.20 (1.18)	3.83 (934)	<0.001	0.25
ASE	2.93 (1.18)	2.79 (1.20)	1.76 (945)	0.079	0.11
CIS *	3.16 (1.31)	2.24 (1.31)	10.84 (946)	<0.001	0.70
SPS *	4.24 (1.04)	3.95 (1.29)	3.84 (895)	<0.001	0.25
SCS *	4.47 (0.81)	3.95 (1.29)	10.79 (796)	<0.001	0.70

* Higher values indicate higher levels of SWB due to reverse coding.

**Table 2:** Centrality Measures.

Variable	Cluster	Strength (Boys)	Strength (Girls)
ES	Internalizing	0.874	0.918
PP	Internalizing	0.605	0.612
CP	Externalizing	0.578	0.695
HI	Externalizing	0.769	0.657
ATS	School Well-Being	0.751	0.908
ATC	School Well-Being	1.162	1.070
ASE	School Well-Being	0.776	0.828
CIS	School Well-Being	0.725	0.641
SPS	School Well-Being	0.712	0.577
SCS	School Well-Being	0.839	1.055

## Data Availability

The dataset and R-Code generated for this study can be found in the Open Science Framework (OSF) under: https://osf.io/mt89e/overview?view_only=ddd74411ed5045b2a3c307139606e84b (accessed on 13 April 2026).

## Abbreviations

The following abbreviations are used in this manuscript:

SWB	School-Related Well-Being
ES	Emotional Symptoms
PP	Peer Problems
CP	Conduct Problems
HI	Hyperactivity/Inattention
ATS	Attitudes Toward School
ATC	Affinity to Class
ASE	Academic Self-Esteem
CIS	Concerns in School
SPS	Social Problems in School
SCS	Somatic Complaints in School
